# Supplementary material for: Infection of Brain Organoids and 2D Cortical Neurons with SARS-CoV-2 Pseudovirus
Source: Viruses. 2020 Sep 8;12(9):1004. doi: 10.3390/v12091004 (PMC7551632; doi:10.3390/v12091004)
Supplement: Supplementary file 1 [file viruses-12-01004-s001.pdf]

viruses

MDPI

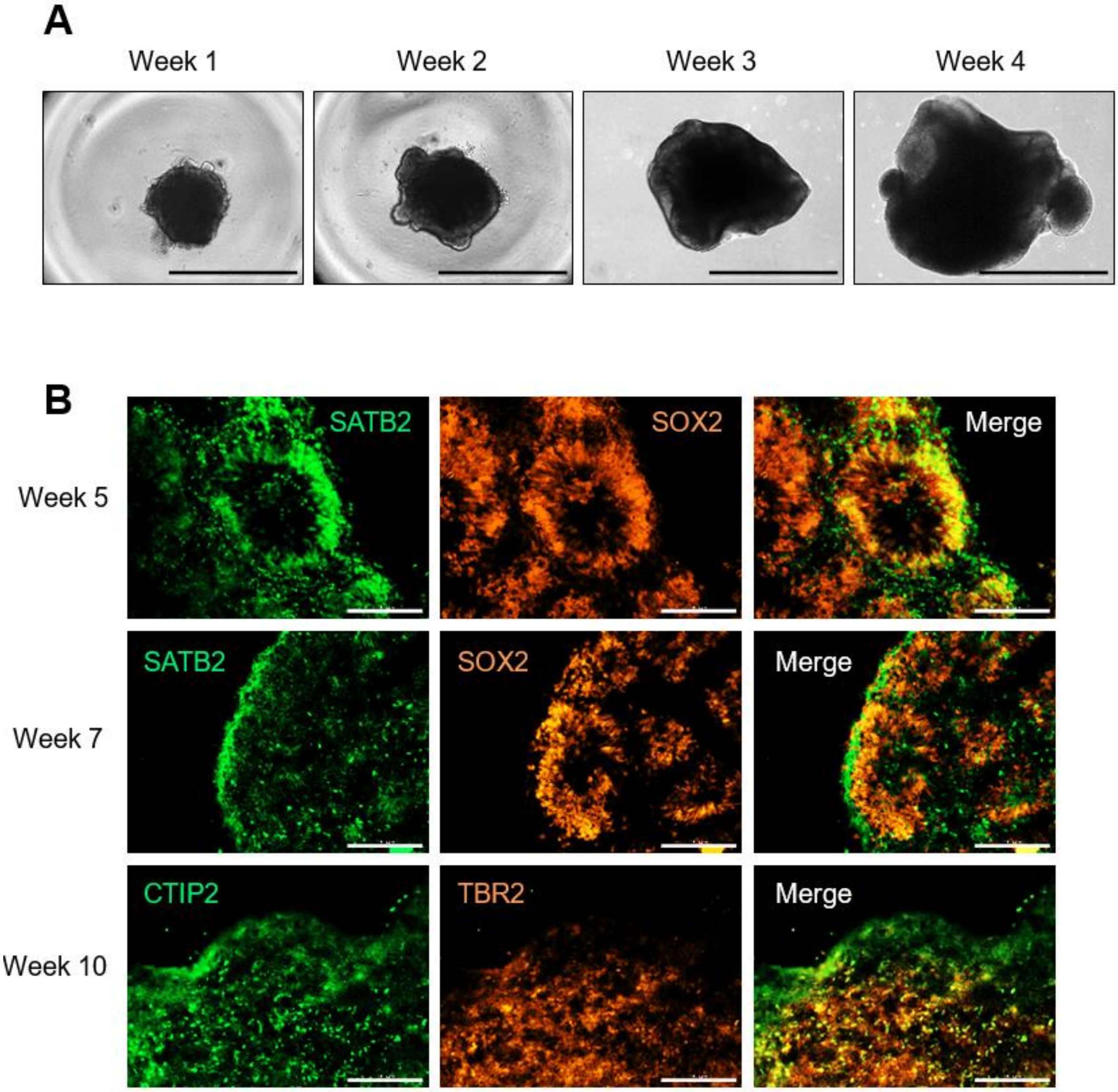

Supplementary Figure 1. Generation of dorsal forebrain organoids from human embryonic stem cells (**A**) Phase contrast images of dorsal forebrain organoids after 1, 2, 3, and 4 weeks of differentiation from H7 cells. Scale bar: 1 mm. (**B**) Immunohistochemistry images of dorsal forebrain organoids after 5, 7, and 10 weeks of differentiation from H7 cells. Scale bar: 100 μm.
